# Home Health Agency Ownership and Quality of Care Outcomes Among Medicare Beneficiaries

**DOI:** 10.1093/geroni/igab046.076

**Published:** 2021-12-17

**Authors:** Rashmita Basu, Huabin Luo, Bei Wu

**Affiliations:** 1 East Carolina University, Greenville, North Carolina, United States; 2 New York University, New York, New York, United States

## Abstract

Medicare restructured home healthcare reimbursement from a cost-basis to a 60- day risk-based prospective payment system (PPS) in 2000 to implement the value-based payment model for home healthcare services. Currently home healthcare market in the U.S. is dominated by the presence of for-profit (FP) agencies instead of being primarily served by not-for-profit (NFP) agencies. Using data from the 2016-2018 OASIS for beneficiaries participated in the Medicare Current Beneficiary Survey (MCBS) (N=6,115), the current study examines whether home health agency ownership status is associated with length of stay (LOS) and discharge outcome Medicare home health care patients. Our first outcome variable is discharge status (modeled via ordered probit) with three categories: discharge to the community, inpatient hospital and other long-term care facilities. The second outcome variable is LOS and two dummy variables LOS ≤ 30 days and LOS ≥ 99 days were modeled via binary probit. The key independent variable was the ownership status of the agency (FP vs. NFP). Patient level covariates includes demographics (age, gender, race/ethnicity, marital status), comorbidity index, agency characteristics (metropolitan statistical area, hospital-based). Patients in FP agencies were 5.1% (p<0.01) less likely to discharge to community, 15.3% (p<0.001) less likely to have LOS ≤ 30 days but 7.5% (p<0.001) more likely to have LOS ≥ 99 higher compared to patients from NFP agencies under the PPS. Our results have important implications for clinicians, patients and healthcare professionals to be cognizant about the influence of agency ownership on the delivery of healthcare services in home healthcare sector.

